# Asymptomatic Pulmonary Embolus in a Patient With a Prior Heart Transplant

**DOI:** 10.7759/cureus.87224

**Published:** 2025-07-03

**Authors:** Addison Smartt, Diego Alegre, Jackie Kelley, James Kelley, Brennan Hand, Douglas Rappaport

**Affiliations:** 1 Emergency Medicine, Mayo Clinic in Arizona, Phoenix, USA; 2 Emergency Medicine, Mayo Clinic in Arizona, Scottsdale, USA; 3 Medicine, University of Arizona College of Medicine, Phoenix, USA; 4 Emergency Medicine, Mayo Clinic Alix School of Medicine, Rochester, USA

**Keywords:** emergency medicine physician, emergency medicine resuscitation, heart transplant, pulmonary embolus, systemic anticoagulation

## Abstract

Heart transplant patients have a significant risk for both deep vein thrombosis (DVT) and pulmonary embolism (PE). This report presents a case of asymptomatic PE in a 63-year-old male patient with a history of orthotropic heart transplant nine years prior. The patient initially presented to the Emergency Department with acute right lower extremity pain and was found to have an extensive DVT involving the femoral vein and extending into the deep veins of the right calf. The patient was admitted to the transplant cardiology inpatient service, managed with a heparin infusion, and eventually transitioned to a direct oral anticoagulant with apixaban and discharged on hospital day 2 without further complications. This case highlights the importance of recognizing the heightened risk of venous thromboembolism in heart transplant patients at any point in their post-transplant course, despite the lack of classic symptoms of PE.

## Introduction

Heart transplant patients are described to be at significant risk for venous thromboembolism (VTE). Previous studies suggest the incidence of VTE events occurs at rates up to six times greater than the general population in the first postoperative year [1]. This is due to a combination of immunosuppression, chronic inflammation, immobility, and endothelial damage in the initial post-transplant period. While studies show the highest rates of VTE post transplant occur within the first year (45.1 episodes per 1000 patient years), there is also some persistent risk beyond that of the initial post-transplant period [2].

Additionally, due to the altered anatomy and physiology inherent to heart transplant recipients, these patients may present atypically, for example, lacking chest pain, making the diagnosis of pulmonary embolism (PE) increasingly challenging. These findings underscore the importance of persistent vigilance and maintaining a high index of suspicion in considering VTE in heart transplant recipients at any point in their post-transplant course. Here, we present a case of PE in a patient with a history of a heart transplant nine years prior, who presented without the classic symptoms of PE.

## Case presentation

A 63-year-old male patient presented to the emergency department with right calf pain and edema. The patient's history included an orthotopic heart transplant secondary to viral cardiomyopathy nine years prior. The patient reported a three-day history of persistent symptoms that had exhibited no significant fluctuations in intensity or character during this period. Symptoms were localized to the patient's right calf. He denied any trauma or inciting event. On review of systems, the patient denied associated chest pain, shortness of breath, fever, chills, hemoptysis, syncope, or palpitations. His past medical history was otherwise significant for one prior deep vein thrombosis (DVT) and PE two years previously, noncompliant with anticoagulation therapy secondary to the prohibitive cost of apixaban, and poorly controlled type II diabetes mellitus. The patient was compliant with his immunosuppressive regimen, which included tacrolimus and mycophenolate. 

**Figure 1:**
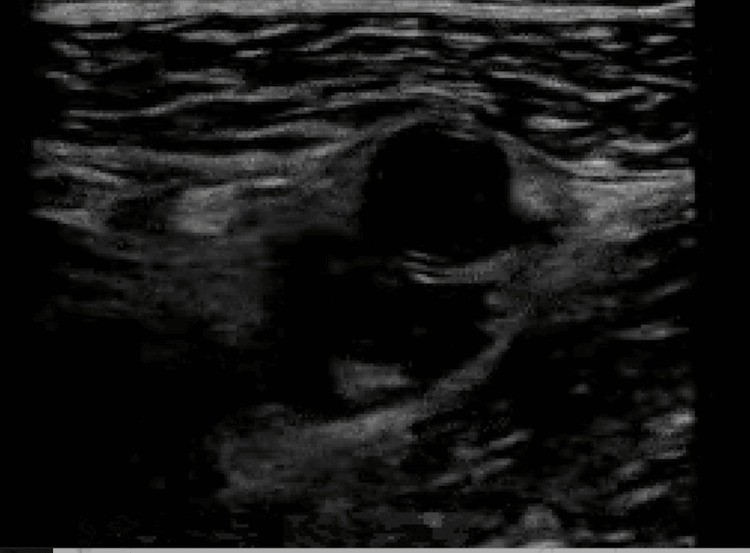
Ultrasound of the right lower extremity demonstrating an acute partially occlusive DVT involving the right mid femoral vein DVT: deep vein thrombosis

Upon initial presentation, the patient was hemodynamically stable, although hypertensive with blood pressure of 150/99 mmHg, heart rate of 102 beats per minute, respiratory rate of 18 breaths per minute, and oxygen saturation of 97% on ambient air. On physical exam, the general impression of the patient was overall good, and the patient did not appear in any acute distress. Cardiopulmonary exam revealed mild tachycardia with clear bilateral lung fields. Examination of the right lower extremity demonstrated right lower leg swelling and pain to palpation of the calf. The exam of the left lower extremity was unremarkable without any evidence of swelling or pain to palpation. A complete blood count and basic metabolic panel were within normal limits. An ultrasound of the right lower extremity demonstrated an acute, partially occlusive DVT involving the right mid-femoral vein extending to the deep calf veins (Figure 1).

**Figure 2:**
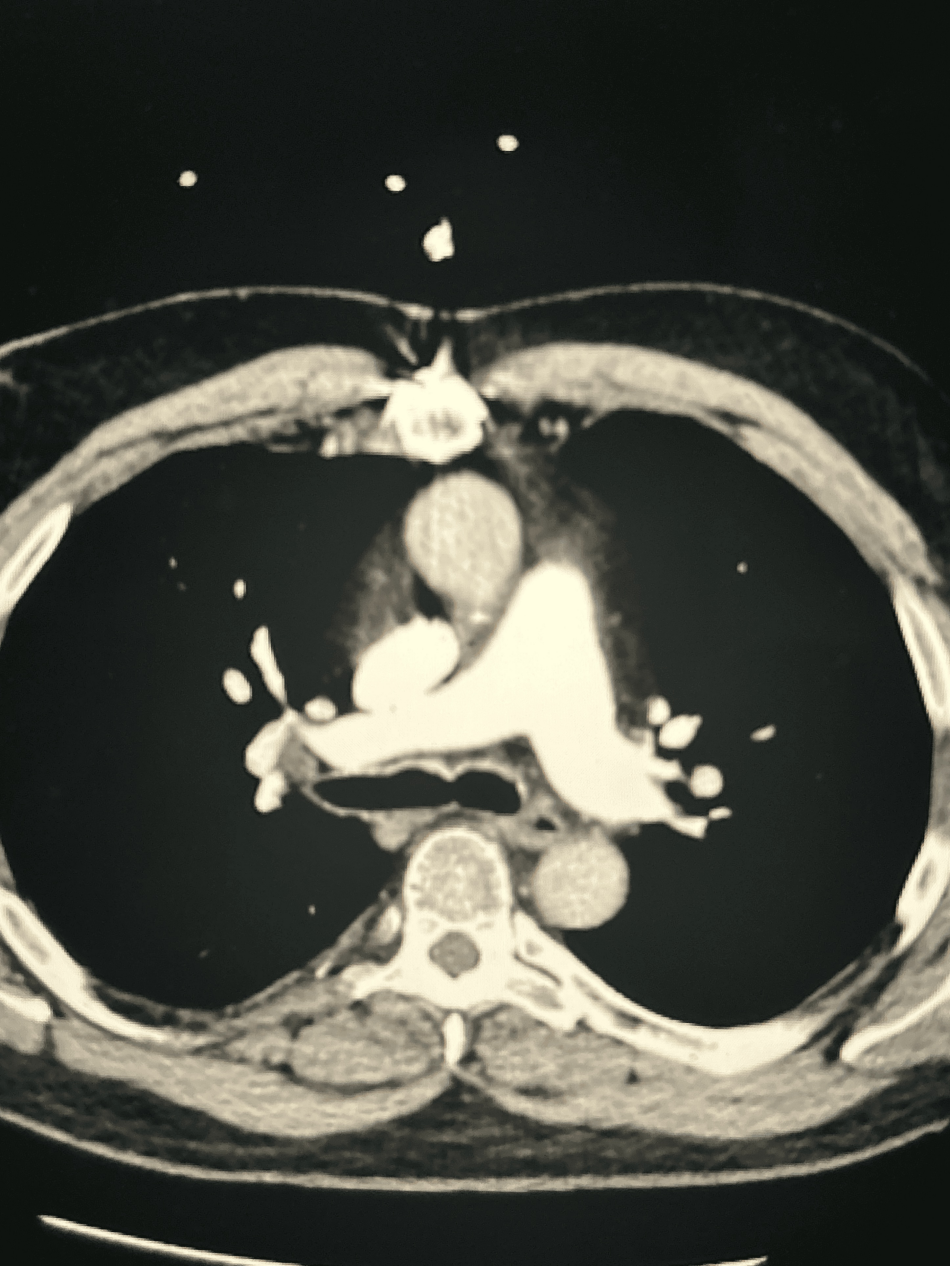
Computed tomography with angiography of the chest demonstrating acute right segmental pulmonary embolus

Given the extent of the DVT, including involvement of the femoral vein, a computed tomography (CT) scan with angiography of the chest was obtained to evaluate for PE (Figure 2). This study demonstrated an acute PE with occlusion of the segmental and subsegmental branches of the right upper and right lower lobes. No evidence of acute right heart strain was found on CT angiogram or subsequent transthoracic echocardiogram. The patient received heparin infusion therapy and was admitted to the cardiology service for further management.

During his admission, the patient was continued on a heparin infusion. On the second day of hospitalization, the patient was discharged without any complications. He was restarted on long-term anticoagulation therapy with apixaban, and the hospital case management team ensured adequate resources to continue therapy without interruption. These interventions included direct discussions with the patient's insurer to guarantee affordable pricing for anticoagulation and regular interval outpatient follow-up to ensure medication compliance. 

## Discussion

This case considers the relationship of an orthotopic heart transplant recipient presenting with a proximal right lower extremity DVT who was ultimately found to have segmental and subsegmental PEs on CT angiography despite the lack of any classic symptoms suggestive of PE, such as chest pain, shortness of breath, or hemoptysis. This case raises several interesting questions specific to patients with prior heart transplantation. 

Patients with newly diagnosed DVT face an increased risk of developing PE [2]. One study found that patients with prior heart transplant faced up to a six-fold increased risk of VTE in the initial one year of transplant [1]. Our patient presented well beyond this initial postoperative period, and it is unclear exactly what the risk of VTE is in this time period. Annually, DVT occurs in one to three out of every 1,000 individuals in the general population, and the likelihood of subsequent PE depends on the thrombus location, other comorbidities, and risk factors [3-7]. 

Our case raises an additional critical question regarding the incidence of asymptomatic PE in patients with newly diagnosed DVT. A systematic review investigating 28 studies found that 32% of patients with DVT also had a silent PE, and the risk of recurrent PE was notably higher in these individuals [7]. In another study by Krutman et al., 52 patients with newly diagnosed DVT were studied, dividing them into two groups based on the location of the clot, proximal versus distal, to assess for the presence of concurrent asymptomatic PE. All patients without respiratory symptoms underwent CT pulmonary angiography. Remarkably, 72% of these asymptomatic patients were found to have a PE regardless of the location of their DVT [8]. These findings suggest that asymptomatic PE in patients with newly developed DVT may be more common than previously recognized, warranting further clinical awareness and possibly routine imaging. As such, in higher-risk patient populations such as heart transplant recipients, silent PE should be considered in those found to have DVTs. 

Ultrasound revealed an acute DVT of the right lower extremity, involving the mid-femoral, popliteal, and distal deep calf venous segments. Despite the findings of Krutman et al. [8], which showed no difference in the presence of PE regardless of DVT location (proximal or distal), we questioned whether the proximal origin of our patient's DVT increased their risk of developing an asymptomatic PE. One study supporting this idea found that asymptomatic PE was present in 42% of patients with proximal DVT compared to 17% in those with distal DVT [6]. Another study reported that the presence of silent PE with proximal DVT occurred in 36% of patients and 13% with distal DVTs [7]. However, these studies are in stark contrast to the previously mentioned Krutman et al.'s study, which found no significant difference in silent PE between proximal and distal DVTs [8]. This discrepancy underscores the complexities of assessing PE risk in patients with newly diagnosed DVT, as other factors may hold influence beyond simply the location of the thrombus. Nevertheless, clinicians must have a high index of suspicion for silent PE in especially high-risk patients with proximal DVTs. 

Additional factors may have contributed further to the development of VTE in this patient, including their history of heart transplant, prior/current history of DVT/PE, male sex, age, immunosuppressive therapy, diabetes, and non-compliance with anticoagulation. In particular, we were interested in investigating the risk of heart transplant patients for developing DVT/PE that extends well beyond their original transplant date, independent of other risk factors. Although the incidence of VTE in heart transplant recipients is well documented in the existing literature [1,9,10], limited data exist on how this risk varies based on the timing of transplantation and how this risk changes over time. 

In considering this, one must first redefine the idea of asymptomatic PE in heart transplant patients, as tachycardia is a common baseline state in this patient population with loss of parasympathetic innervation post transplantation [11]. Indeed, this patient did present with tachycardia with a heart rate of 102 beats per minute, which again is an expected finding in this patient population. Furthermore, chest pain may be questionably absent due to the denervated transplant as well. As mentioned previously, our patient did not have the classic symptoms associated with PE, such as dyspnea, chest pain, or hemoptysis. A review regarding early and late reinnervation noted that reinnervation appears in some but not all transplant recipients and also concluded that even after 10 years post transplant, reinnervation was not present in all patients [11]. Such findings suggest that classic signs and symptoms of PE may remain absent beyond the initial transplant period. 

A review of the literature on VTE in heart transplant patients, as mentioned previously, demonstrates an association between VTE and orthotopic heart transplant [9]. Our patient had risks beyond heart transplant to include prior DVT/PE and the presence of a proximal DVT, but we cannot conclude specifically whether his heart transplant history or the immunosuppressive drugs he was taking were the ultimate etiology of his DVT/PE. Nevertheless, it seems that clinicians should consider heart transplant as a risk for VTE of unknown duration well beyond the one-year post-transplant period. We can conclude that patients with newly diagnosed DVT are at significant risk for the presence of asymptomatic PE, regardless of other contributing factors. The loss of cardiac innervation might further complicate the clinical presentation, masking typical symptoms and signs of PE, and causing some confusion in the interpretation of vital sign abnormalities in this patient population as well. This case underscores the critical need for heightened vigilance and proactive consideration of PE in this patient population. Notably, a gap persists in the existing literature regarding the optimal management of elevated PE risk among heart transplant recipients. Further research is warranted to address this deficiency and to develop evidence-based screening protocols aimed at the timely prevention and diagnosis of PE in this high-risk cohort.

## Conclusions

This unique clinical case highlights the potentially complicated presentation of PE in heart transplant recipients as well as the heightened risk of PE in patients with extensive and proximal DVTs. Even in the absence of classic symptoms of PE, patients with proximal and extensive DVTs are at risk for PE. Given that heart transplant patients specifically have been demonstrated to have an increased incidence of PE, particularly in the initial post-transplant period, clinicians should maintain a heightened concern for the possibility of DVT and asymptomatic PE in this patient population even beyond the initial one-year post-transplantation period. Furthermore, understanding the unique physiology that is inherent to patients post cardiac transplantation is essential when evaluating this specific patient population.
